# The placenta goes viral: Retroviruses control gene expression in pregnancy

**DOI:** 10.1371/journal.pbio.3000028

**Published:** 2018-10-09

**Authors:** Edward B. Chuong

**Affiliations:** BioFrontiers Institute, Department of Molecular, Cellular, and Developmental Biology, University of Colorado, Boulder, Colorado, United States of America

## Abstract

The co-option of endogenous retroviruses (ERVs) is increasingly recognized as a recurrent theme in placental biology, which has far-reaching implications for our understanding of mammalian evolution and reproductive health. Most research in this area has focused on ERV-derived proteins, which have been repeatedly co-opted to promote cell–cell fusion and immune modulation in the placenta. ERVs also harbor regulatory sequences that can potentially control placental gene expression, but there has been limited evidence to support this role. In a recent study, Dunn-Fletcher and colleagues discover a striking example of an ERV-derived enhancer element that has been co-opted to regulate a gene important for human pregnancy. Using genomic and experimental approaches, they firmly establish that a primate-specific ERV functions as a placenta-specific enhancer for *corticotropin-releasing hormone* (*CRH*), a hormone linked to the control of birth timing in humans. Their findings implicate an extensive yet understudied role for retroviruses in shaping the evolution of placental gene regulatory networks.

## The rapidly evolving placenta

The ancestral form of our placenta emerged roughly 130 million years ago, marking a key evolutionary innovation that enabled live birth in mammals [1]. In mammalian development, the fetal placenta is the first organ to form and is responsible for anchoring the embryo to the uterus and mediating physiological exchange with the mother. The placenta sustains the fetus throughout pregnancy, and defects in placentation are at the root of many pregnancy complications. Yet despite its significance for evolution, development, and reproductive health, the placenta is arguably the least understood of all mammalian organs.

One of the unique challenges to studying the placenta is the fact that it exhibits unexpectedly wide variation in form and function across species, despite performing a conserved role supporting fetal development. The mammalian placenta shows great diversity across species in its morphology and tissue organization, mechanisms of implantation and invasion, and physiological regulation [2,3]. Even trophoblast cells, the cellular building blocks of the placenta, bear little morphological or molecular resemblance across species [4]. Current theories suggest that life history changes and/or parent–offspring conflicts over maternal resources promoted rapid evolution of the placenta [5,6]. The diverse array of placental shapes and sizes has complicated efforts to model human placentation in other animals but also underscores its unusual biology and evolutionary history [7].

What fueled the explosive evolutionary diversification of the mammalian placenta? Addressing this question is crucial for understanding mammalian reproductive biology and human-specific conditions of pregnancy [1,8]. Intriguingly, a mounting body of evidence suggests that the evolution of the placenta had significant assistance from ancient retroviruses.

## Placenta's little helpers

Retroviruses, which include contemporary viruses such as human immunodeficiency virus (HIV) and human T-lymphotropic virus (HTLV), have been infecting vertebrates for over 450 million years [9]. A key step in the retrovirus lifecycle is retrotransposition, in which the RNA-based virus genome is reverse transcribed and integrated into the DNA of the host cell. Occasional integrations into the germline genomes of egg or sperm cells have the potential to become endogenous retroviruses (ERVs) that become fixtures in the host genome. Genome-sequencing projects of many species have revealed that ERVs have a ubiquitous presence in vertebrate genomes, constituting over 8% of the human genome [10]. Most ERVs are considered fossilized relics that can no longer replicate or encode functional viruses, but the occasional insertion may prove beneficial for the host and become co-opted for a cellular role.

Since the discovery of ERVs decades ago, a number of retroviral proteins have been identified that have been co-opted to perform a wide range of biological functions [11]. Notably, there is an apparent propensity for ERVs to acquire new roles in the placenta [12]. One of the most iconic examples of retrovirus "domestication" is the gene *Syncytin-1*, which originates from a retroviral envelope gene. In primates, *Syncytin-1* was repurposed for the development of a multinucleate tissue layer known as the syncytiotrophoblast, which separates maternal and fetal bloodstreams in the placenta [13]. Remarkably, *Syncytin*-like retroviral proteins have been reported to be expressed in the placentas of nearly all mammals, yet *Syncytins* in different lineages derive from at least 10 independent infections by unrelated retroviruses [14]. These findings have led to speculation that the co-option of unrelated ERVs in different species was a driving force underlying the evolutionary diversification of the placenta [15].

## Rewiring the placenta

Functional studies have revealed that ERV-derived proteins tend to be co-opted for three roles in the placenta: mediating cell–cell fusion to form a multinucleate barrier, suppressing maternal immunity, and protecting the fetus from exogenous viruses [12,16]. However, recent evidence suggests that ERVs may play an even more pervasive role in placenta evolution as noncoding regulatory elements [17].

**Fig 1:**
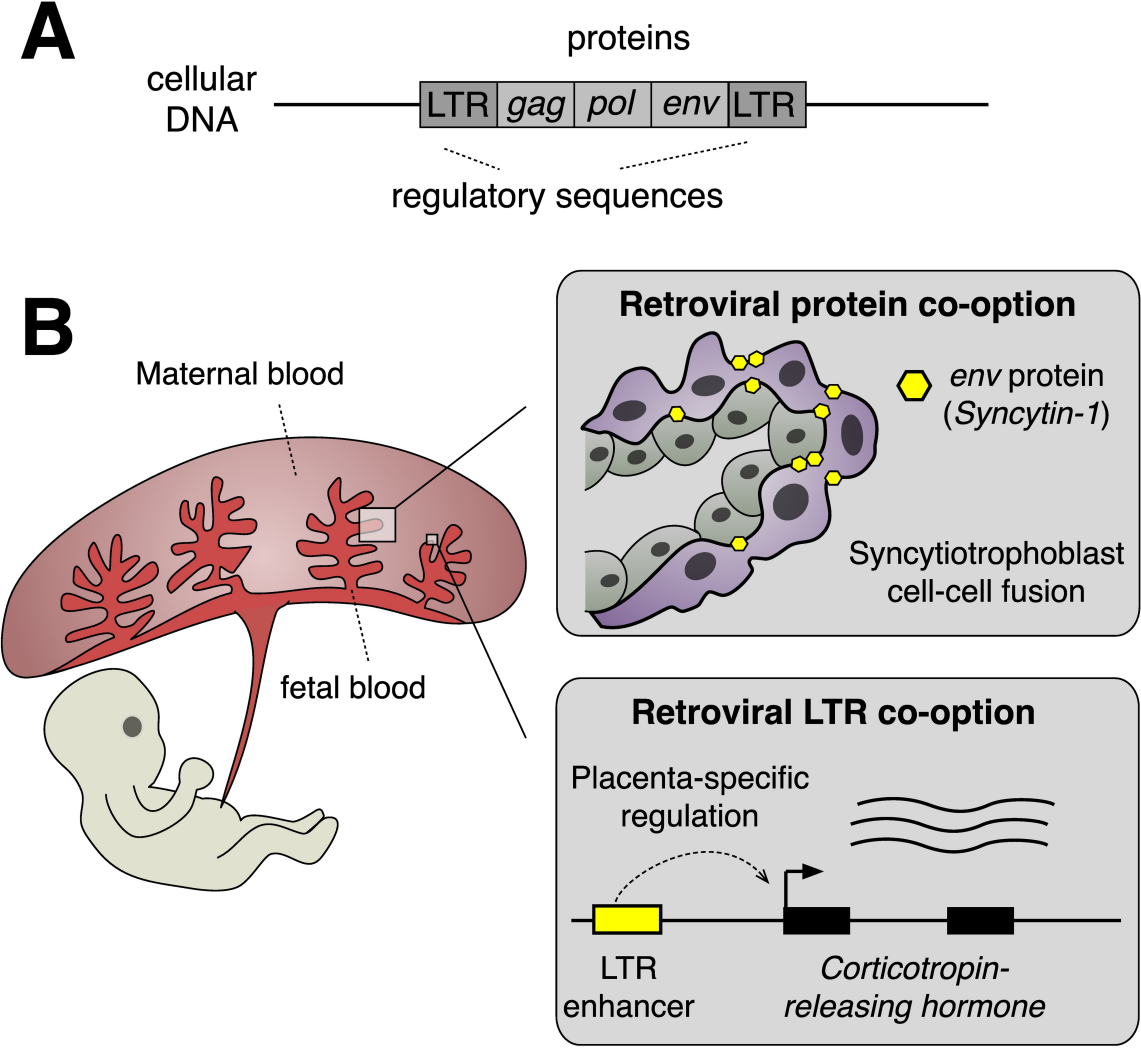
A) Schematic of an endogenous retrovirus upon integration in the host genome. B) Examples of retrovirus protein-coding [13] and regulatory sequence [24] co-option in the placenta. LTR, long terminal repeat.

The idea that repetitive elements such as ERVs may serve as basic components of gene regulatory networks can be traced back over a half-century to the foundational work of Barbara McClintock, Eric Davidson, and Roy Britten [18,19]. ERVs often include long terminal repeat (LTR) viral promoter sequences, which can act as cellular promoters or enhancers to modulate the expression of nearby host genes (Fig 1A). The actual contribution of these elements to host gene expression has remained mostly obscure due to their repetitive and noncoding nature, but technological advances such as chromatin immunoprecipitation followed by sequencing (ChIP-Seq) have helped to cast these elements back into the spotlight. Large-scale analyses of regulatory elements have revealed that ERVs constitute a surprisingly substantial fraction of cell type-specific regulatory elements in mammalian cells, particularly embryonic stem cells and placental cells [20–22]. For example, in mouse trophoblast stem cells, over 30% of predicted enhancer elements marked by the occupancy of core placental transcription factors are derived from copies of a single ERV family [23].

While genomic studies point to ERVs as an abundant source of placenta-specific regulatory elements, their biological relevance to pregnancy remains largely unexplored. Given their retroviral origins, ERVs are not expected to have any benefit for the host, and some may even exhibit pathological activity. Therefore, without further experimental support, it remains unclear whether ERV-derived regulatory activity is actually relevant for placental function.

## Regulation of birth timing

In a study published in this issue of *PLOS Biology* [24], Dunn-Fletcher and colleagues present strong evidence that ERVs can have important gene regulatory activities in pregnancy. The authors originally set out to study the regulation of *corticotropin-releasing hormone* (*CRH*), which encodes a peptide hormone produced in massive quantities in the human placenta. Placental *CRH* levels undergo exponential increases throughout gestation, and misregulation of *CRH* is associated with premature and post-term birth [25]. Determining how *CRH* is regulated is important for understanding the molecular control of birth timing but is complicated by the fact that placental expression of *CRH* is unique to primates. In all other mammals, *CRH* is expressed primarily in the hypothalamus and the brain [26].

The authors hypothesized that placental expression of human *CRH* is determined by primate-specific regulatory sequence near the gene. Through their examination of the noncoding genomic region surrounding human *CRH*, they identify a primate-specific ERV insertion located 2 kilobase pairs upstream of the gene. The authors find little transcriptomic evidence for spliced *CRH* transcripts originating from the ERV, which suggests that the element may act as an enhancer rather than a promoter. The insertion is an LTR sequence that originates from an ancient retrovirus named transposon-like human element 1B (THE1B), which invaded the anthropoid primate lineage approximately 50 million years ago and dispersed roughly 20,000 elements in the genome before eventually becoming inactivated by mutations. On a genome-wide level, genes located near THE1B-derived LTR elements (within 20 kb) tend to show placenta-specific expression patterns, suggesting that THE1B elements are an abundant source of placenta-specific enhancers in primate genomes.

The authors next seek to experimentally investigate whether the THE1B element is indeed required for placental *CRH* expression. Given that placental *CRH* expression is unique to primates and the THE1B element is a primate-specific ERV, it would seem futile to model the regulation of this locus in mice or any other nonprimate animal. The authors sidestep this obstacle by creating mice with "humanized" placentas, with respect to the *CRH* locus. They generate transgenic mice harboring a stably integrated 180 kilobase pair-long bacterial artificial chromosome (BAC) encompassing the human *CRH* gene and upstream THE1B element. Mice typically only show *CRH* expression in the hypothalamus, but the transgenic mice showed robust and specific expression of human *CRH* in the hypothalamus and placenta, confirming their usefulness as a model for *CRH* regulation. Remarkably, mice expressing human *CRH* in the placenta were born on average 15 hours later that control mice, which coincides with the proposed role for *CRH* in regulating the timing of human birth.

To test whether the THE1B LTR element is required for expression of human *CRH* in the placenta, the authors use clustered regularly interspaced short palindromic repeats (CRISPR) to delete the element from the human BAC sequences in the transgenic mice. In this second round of mutants, human *CRH* was still expressed in the hypothalamus, but its expression in the placenta was eliminated. Strikingly, deletion of the THE1B element fully rescued gestation length back to control levels. These experiments confirm that the THE1B element functions as a placenta-specific enhancer of *CRH* and suggest that the co-option of this retroviral element was an important step in the evolution of birth timing in primate pregnancy.

Finally, the authors sought to determine the upstream transcription factors responsible for activating THE1B enhancer activity in the placenta. By analyzing transcriptomes generated from placentas of both human and rhesus macaque, they identify *distal-less homeobox 3* (*DLX3*) as a candidate factor, which is coexpressed with *CRH*, has a predicted binding site in the 5′ region of the THE1B insertion and is known to be required for placental development. The authors confirm that DLX3 physically interacts with the THE1B element using electrophoretic mobility shift assays and chromatin immunoprecipitation experiments in human placental tissue. Synthesizing their findings, the authors propose a model where the *CRH* gene is transcriptionally activated in the placenta through the binding of DLX3 to the THE1B-derived enhancer, which ultimately influences birth timing in pregnancy.

## Outlook

The study by Dunn-Fletcher and colleagues demonstrates that ERV LTRs can have biologically significant gene regulatory activities in the placenta and offers a new clue into the molecular basis of human birth timing. Their findings come with the caveat that the phenotypic effects of *CRH* expression were observed in transgenic mice, which do not normally express *CRH* in the placenta. However, without generating transgenic primates, the approach used in this study remains the most practical option for experimentally dissecting primate-specific gene regulation in the placenta.

Several outstanding questions remain. First, how generally important are ERVs for placental gene expression? There is now strong evidence that ERV-derived proteins play an important role in placental development in many mammals, but whether ERV-derived regulatory elements play similarly significant roles remains unknown. In addition to the enhancer uncovered by this study, ERVs have also contributed a placenta-specific promoter for the human *cytochrome P450 family 19 subfamily A member 1* (*CYP19*) gene and an enhancer for the human *major histocompatibility complex*, *class I*, *G* (*HLA-G*) gene [27,28]. Aside from these few examples, the specific effects for most ERV-derived regulatory elements remain largely unknown and require experimental verification. As more potentially regulatory ERVs are experimentally characterized, we will eventually obtain a better understanding of their relative contribution to placenta evolution.

Second, why is ERV transcriptional activity apparently elevated in placental cells? The placenta has long been observed to be a hotbed of ERV expression compared to the embryo, but the reasons for this remain poorly understood [29,30]. One explanation is that placenta-specific activity originally evolved as a parasitic feature of the retrovirus LTR. Viral expression in the placenta can facilitate maternal–fetal transmission of retroviruses and can theoretically also permit ERVs to invade the germlines of other offspring [31]. Consequently, ERVs may have repeatedly evolved LTR promoter sequences with strong placenta-specific activity in order to drive their selfish replication. Another possibility is that the unique epigenetic environment of extraembryonic trophoblast cells may be generally more permissive to ERV transcription compared to embryonic cells [30,32–34]. This suggests that placental cells are intrinsically more tolerant to ERV activity (perhaps due to their transient nature) or that ERV transcriptional activity in the placenta somehow provides a general fitness benefit to the fetus and/or mother. Whatever the reason, elevated levels of ERV transcription in the placenta likely facilitated their recurrent co-option for functions in the mammalian placenta.

Finally, how do regulatory ERVs influence susceptibility to pregnancy-related complications? Misexpression of ERV-derived proteins such as *Syncytin* has been associated with human pregnancy disorders such as pre-eclampsia [35]. However, the potential impact of noncoding LTRs on placental diseases remains largely unexplored. Misregulation of the THE1B enhancer at the *CRH* locus, due to noncoding genomic mutations or misregulation of upstream regulators such as DLX3, may be a contributing factor to premature or post-term birth. The co-option of LTRs as enhancer elements may also pose a conundrum for the cell and its intrinsic transposon silencing pathways, which must be tuned to permit the activity of beneficial ERVs while repressing potentially deleterious ones. Environmental or stochastic perturbations of these pathways during pregnancy may disrupt or activate genes with wide-ranging pathological effects.

To conclude, the study by Dunn-Fletcher and colleagues adds to a growing body of evidence supporting a remarkable evolutionary relationship between retroviruses and the placenta. A picture is now emerging in which important features of placentation are often reliant on proteins and regulatory sequences co-opted from ancient retroviruses (Fig 1B). It is tempting to speculate that human pregnancy would be very different—perhaps even nonexistent—were it not for eons of retroviral pandemics afflicting our evolutionary ancestors. Intriguingly, recent studies have reported ERV expression in the placenta-like organs of some live-bearing species of fish and reptiles, which suggests that retroviruses may have widely convergent roles promoting the evolution of live birth in their hosts [36,37]. Future studies such as Dunn-Fletcher and colleagues investigating ERV function in the placentas of humans and other animals will be important to uncover the full extent to which retroviruses have shaped the extraordinary biology and evolution of the placenta.

## Abbreviations

BAC: bacterial artificial chromosome
ChIP-Seq: chromatin immunoprecipitation followed by sequencing
CRH: corticotropin-releasing hormone
CRISPR: clustered regularly interspaced short palindromic repeats
CYP19: cytochrome P450 family 19 subfamily A member 1
DXL3: distal-less homeobox 3
ERV: endogenous retrovirus
HIV: human immunodeficiency virus
HLA-G: major histocompatibility complex, class I, G
HTLV: human T-lymphotropic virus
LTR: long terminal repeat
THE1B: transposon-like human element 1B
